# Zincology: Decoding Homeostasis to Unveil Disease Mechanisms

**DOI:** 10.34133/research.1080

**Published:** 2026-01-12

**Authors:** Tianyi Wang, Junxia Min, Fudi Wang

**Affiliations:** ^1^ School of Public Health, Hengyang Medical School, University of South China, Hengyang 421001, China.; ^2^ The Second Affiliated Hospital, School of Public Health, Zhejiang University School of Medicine, Hangzhou 310058, China.; ^3^ The First Affiliated Hospital, Institute of Translational Medicine, Zhejiang Key Laboratory of Frontier Medical Research on Cancer Metabolism, Zhejiang University School of Medicine, Hangzhou 310058, China.; ^4^ Global Innovation Institute of Element Science (GIIES-JLU), The First Hospital of Jilin University, Changchun 130021, China.

## Abstract

Zincology, the rigorous cross-disciplinary study of zinc metabolism and its multifaceted biological and applied roles in health and disease, delivers a transformative framework for resolving long-standing uncertainties in biomedical research. As a central regulator of cellular function and a versatile element in applied fields, zinc dyshomeostasis underpins diverse high-burden pathologies, including renal, metabolic, cardiovascular, and neurodegenerative disorders, yet its mechanisms remain fragmented across disciplines. This Perspective marks the first formal definition of Zincology as a distinct cross-disciplinary discipline, clarifying zinc’s context-dependent dual role: systemic deficiency exacerbates biological injury through compromised antioxidant defense and inflammation, while local excess drives pathogenesis—exemplified by the Zn–protein kinase B (AKT)–forkhead box protein O1 (FOXO1)–glucose-6-phosphatase catalytic subunit (G6PC) axis in kidney disease. We synthesize Zincology’s core principles to integrate systemic and local zinc metabolism, dissect its pathogenic role in the aforementioned disorders, and outline cross-disciplinary research directions with implications extending beyond biomedicine. Leveraging Zincology’s multidisciplinary rigor, we establish zinc homeostasis as a unifying framework for deciphering disease mechanisms, with kidney disease serving as a paradigmatic model to validate its core tenets, bridging fragmented basic, clinical, industrial, and environmental research to address global critical unmet medical and societal needs. Notably, Zincology extends beyond biomedicine to encompass engineering, ecology, and other frontier fields, representing a comprehensive cross-disciplinary system that links basic science with diverse applied domains.

## Background

Zinc, an essential micronutrient [1], underpins hundreds of enzymatic reactions, cell signaling pathways, hematopoiesis [2], and immune processes [3,4]—yet its role in kidney disease has long been oversimplified. The kidney, as the primary organ regulating systemic zinc balance, reabsorbs ~95% of filtered zinc to prevent deficiency and excretes excess to avoid toxicity [1]. Early research focused narrowly on systemic zinc deficiency, which induces oxidative stress, hypertension, and subsequent kidney damage. However, the pathogenic impact of renal local zinc metabolism remained unaddressed, leaving key questions about zinc’s role in acute kidney injury (AKI) and chronic kidney disease (CKD) unresolved.

**Fig. 1.**
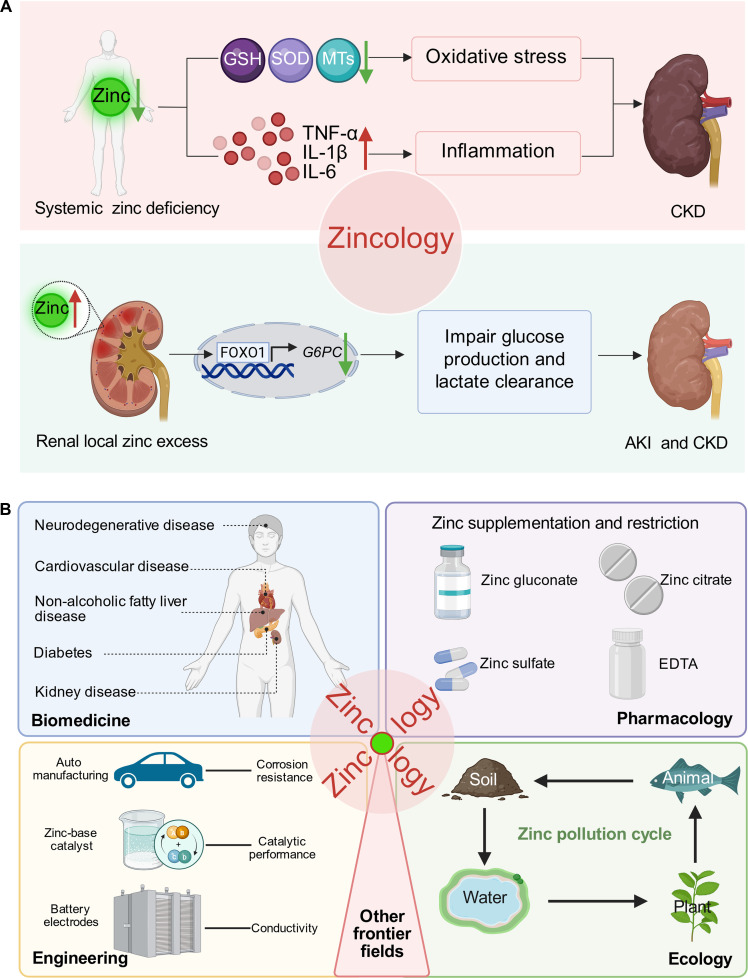
Zincology: From systemic and local zinc dysregulation to cross-disciplinary integration. (A) Zincology clarifies zinc’s context-dependent roles in kidney disease by linking systemic/renal zinc homeostasis to pathological outcomes. Top panel: Systemic zinc deficiency exacerbates kidney injury. In line with Zincology’s “deficiency-induced dysfunction” principle, insufficient systemic zinc impairs antioxidant activity (reduced MT/SOD) and activates pro-inflammatory pathways (NF-κB/MAPK), increasing renal ROS production, promoting tubular apoptosis, and accelerating fibrosis—worsening AKI/CKD (supported by clinical links between low serum zinc and faster eGFR decline). Bottom panel: Renal local zinc excess drives kidney disease. Guided by Zincology’s “local dyshomeostasis-induced toxicity” principle, excess renal zinc (via impaired SLC30A1 efflux or increased SLC39A8 influx) disrupts the Zn–AKT–FOXO1–G6PC axis, reducing G6PC-mediated gluconeogenesis, causing lactate accumulation, and exacerbating tubular necrosis/fibrosis—effects reversed by zinc chelators (e.g., EDTA) in preclinical models. (B) The cross-disciplinary application framework of Zincology. This framework integrates 5 core fields to reflect Zincology’s interdisciplinary scope, with key applications as follows: Biomedicine: Connecting zinc homeostasis to major diseases, including kidney disease, diabetes, cardiovascular disease, neurodegenerative disease, and metabolic-associated fatty liver disease. Pharmacology: Delivering zinc supplementation (e.g., zinc gluconate, zinc sulfate, and zinc citrate) for zinc-deficient populations, using zinc chelators (e.g., EDTA) for populations with zinc excess, and developing kidney-specific zinc regulators. Ecology: Managing the zinc cycle across soil, water, plants, and animals—such as mitigating zinc pollution and maintaining zinc homeostasis in ecosystems. Engineering: Applying zinc in automotive manufacturing, battery electrodes, and zinc-based catalysts. Other frontier fields: Encompassing interdisciplinary innovations, including zinc in quantum materials research, zinc-based energy storage systems, and zinc-mediated environmental remediation technologies. This multi-field system embodies the cross-disciplinary nature of Zincology, the first formally defined discipline dedicated to systematic zinc research. Figure created using BioRender.

Zincology—first referenced in foundational zinc biology work [5]—has evolved beyond single-discipline approaches to become a comprehensive field integrating biochemistry, genetics, physiology, and clinical medicine. Its core tenets—zinc homeostasis as a multi-organ network, zinc’s context-dependent activity, and dyshomeostasis as a causal disease factor—provide the clarity needed to advance renal zinc research. Our recent work [1], which identified renal local zinc excess as a driver of AKI/CKD, exemplifies Zincology’s value in bridging basic and translational nephrology. This Perspective builds on this work to position Zincology as a key framework for revolutionizing research into major diseases, while also acknowledging its inherent cross-disciplinary nature that spans life sciences, engineering, ecology and beyond, with broad applications across multiple fields (Fig. 1.).

## Zincology: A Multidisciplinary Foundation for Zinc Homeostasis Research

Zincology transcends traditional boundaries to dissect the complexity of zinc homeostasis, recognizing that zinc’s biological role is not isolated but part of a dynamic, interconnected system. At its core, Zincology emphasizes rigor in linking molecular mechanisms (e.g., transporters and storage proteins) to organ-level function and clinical outcomes—critical for understanding zinc–disease interactions.

Systemic zinc balance relies on a conserved network orchestrated by 2 transporter families and intracellular buffers: the ZIP/SLC39A family (e.g., SLC39A8/ZIP8) mediates zinc influx, the SLC30A/ZnT family (e.g., SLC30A1/ZnT1 [6]) drives efflux, and metallothionein (MT1/MT2) sequesters free zinc to prevent toxicity or deficiency.

As a cross-disciplinary field, Zincology also integrates genetics to explain interindividual differences. The SLC39A8 A391T variant (rs13107325), a loss-of-function mutation that reduces renal zinc uptake, protects against 12 distinct kidney conditions [1]—a finding that underscores Zincology’s role in identifying novel therapeutic targets for metal ion-related pathologies.

## Zincology in Kidney Physiology: Regulation and Function

Within the kidney, Zincology clarifies 2 interdependent roles: the kidney as a regulator of systemic zinc homeostasis, and zinc as an essential mediator of renal function.

The kidney’s role in systemic balance embodies Zincology’s “local–global integration” principle. Proximal tubule epithelial cells express high levels of SLC39A8 (brush border) and SLC30A1 (basolateral membrane), mediating ~95% of filtered zinc reabsorption [1]. Notably, both SLC39A8 and SLC30A1 are multifunctional metal transporters: SLC39A8 can also transport Fe^2+^ and Mn^2+^ [1], while SLC30A1 additionally facilitates Cu^2+^ transport [7]. Whether zinc exerts synergistic effects with other metal ions (e.g., copper, iron, and manganese) during the progression of kidney disease warrants further investigation.

Zinc, in turn, supports renal function via Zincology’s “context-dependent activity” tenet. It acts as a cofactor for Na^+^-K^+^-ATPase or constitutes a core component of metalloenzymes that maintain renal homeostasis. Zinc deficiency reduces MT expression, resulting in increasing oxidative stress and tubular apoptosis [8], while physiological concentrations balance inflammation and metabolism to preserve renal structural and functional integrity [9].

## Zinc Dyshomeostasis in Kidney Disease: A Zincology Perspective

Zincology resolves zinc’s dual role in kidney disease, distinguishing between systemic deficiency and renal local excess as distinct but equally impactful drivers of injury (Fig. 1).

Aligned with Zincology’s “deficiency-induced dysfunction” framework, systemic zinc deficiency exacerbates renal injury by impairing antioxidant defense and activating NF-κB/MAPK pro-inflammatory pathways, leading to tubular damage and interstitial fibrosis. Notably, fast zinc signaling and late zinc signaling play distinct roles in kidney disease progression: fast zinc signaling is induced by acute stress within seconds [10], limiting initial injury via antioxidant, anti-inflammation, and anti-apoptotic effects; late zinc signaling systematically modulates cellular zinc homeostasis by altering zinc transporter expression [10], thereby influencing long-term CKD progression.

Renal local zinc excess, uncovered via Zincology-guided research, represents a novel pathogenic mechanism. Our recent work [1] shows that kidney-specific SLC39A8 knockout (KKO) mice (reduced zinc uptake) exhibit milder AKI (ischemia–reperfusion and rhabdomyolysis) and CKD (ureteral obstruction and 5/6 nephrectomy), with lower serum creatinine/blood urea nitrogen (BUN) levels and reduced renal fibrosis. Conversely, SLC30A1 knockout (Z-KKO) mice (impaired zinc efflux) accumulate renal zinc and display severe renal injury. Mechanistically, excess zinc activates protein kinase B (AKT), phosphorylates forkhead box protein O1 (FOXO1) to inhibit its nuclear translocation, and down-regulates glucose-6-phosphatase catalytic subunit (G6PC, a gluconeogenic enzyme)—leading to lactate accumulation, a well-characterized driver of tubular injury [1]. This Zn–AKT–FOXO1–G6PC axis highlights how local dyshomeostasis disrupts renal function independent of systemic zinc status.

## Translational Zincology: Targeted Strategies for Kidney Disease

Zincology’s ultimate value lies in translating mechanistic insights into clinical therapies. Three strategies align with its rigor and cross-disciplinary focus: (a) Precision nutritional management, guided by Zincology’s “context-dependent regulation” principle, emphasizes individualized zinc intake. (b) Kidney-specific zinc modulators address the limitations of nontargeted interventions: EDTA, a zinc chelator, ameliorates AKI/CKD in preclinical murine models [1], though its broad-spectrum metal chelating properties raise questions about the contribution of other metal ions to this effect, which requires further experimental validation. (c) Transporter modulators—*SLC39A8* siRNA and SLC30A1 agonists restore local zinc homeostasis without disrupting systemic balance [1].

## Discussion

Zincology’s core principles, validated through our prior kidney disease research [1], provide a scalable framework to interpret zinc dyshomeostasis in other major diseases. While renal pathologies have been the focus of Zincology’s initial translational exploration, its tenets are broadly applicable to high-burden disorders with analogous mechanisms (Fig. 1).

### Metabolic diseases

Zincology extends to metabolic disorders [11,12], diabetes, and metabolic-associated fatty liver disease, which are characterized by zinc dyshomeostasis (systemic deficiency or liver-specific excess) that drives disease pathogenesis [13–16]. These patterns mirror renal zinc dyshomeostasis, unifying fragmented research across metabolic pathologies.

### Cardiovascular disease

Cardiovascular disease, the leading global cause of mortality, exhibits zinc-related vascular dysfunction [17]: Endothelial zinc deficiency promotes atherogenesis [18], while macrophage zinc excess within atherosclerotic plaques amplifies inflammatory responses [19]. Zincology’s “context-dependent role” framework integrates these disparate observations into a cohesive mechanistic model.

### Neurodegenerative disease

Alzheimer’s disease involves synaptic and mitochondrial zinc dysregulation, which drives amyloid-β aggregation and neuronal apoptosis [20]. Zincology unifies these scattered findings to establish zinc homeostasis as a central node in neurodegenerative pathogenesis.

## Conclusion

Zincology, as a rigorous, multidisciplinary discipline, has redefined our understanding of zinc–kidney interactions and, in doing so, uncovered a universal framework for decoding zinc’s role in diverse diseases. It resolves zinc’s dual role in kidney disease and identifies targeted translational strategies, while offering actionable mechanistic insights for metabolic, cardiovascular, and neurodegenerative pathologies. Our prior study [1] provides proof of concept for Zincology’s clinical relevance, with future expansion to other renal pathologies. By leveraging Zincology’s integration of biochemistry, genetics, and clinical science, we can develop precision therapies for diseases affecting billions of people worldwide—advancing zinc biology, translational medicine, and related applied fields.
